# Beta-2 adrenergic receptors increase TREG cell suppression in an OVA-induced allergic asthma mouse model when mice are moderate aerobically exercised

**DOI:** 10.1186/s12865-018-0244-1

**Published:** 2018-02-17

**Authors:** Kari J. Dugger, Taylor Chrisman, Sarah L. Sayner, Parker Chastain, Kacie Watson, Robert Estes

**Affiliations:** 1 Department of Biomedical Sciences, College of Allied Health; University of South Alabama, 5721 USA Dr. N, HAHN 4021, Mobile, 36688 AL USA; 2 Department of Physiology and Cell Biology, Center for Lung Biology, College of Medicine, University of South Alabama, Mobile, 36688 AL USA; 3 Department of Clinical and Diagnostic Sciences, Biomedical Sciences, School of Health Professions, University of Alabama at Birmingham, 1716 9th Ave S, SHPB 472, Birmingham, 35294 AL USA

**Keywords:** Beta-2 adrenergic receptor, Exercise, TREG cell, cAMP

## Abstract

**Background:**

The potency of T regulatory (TREG) cells to inhibit T helper (Th)-driven immune cell responses has been linked to increased intracellular cyclic-AMP (cAMP) levels of TREG cells. In an ovalbumin (OVA)-driven allergic asthma mouse model, moderate aerobic exercise increases TREG cell function in a contact-dependent manner that leads to a significant reduction in chronic inflammation and restoration of lung function. However, the mechanism, whereby exercise increases TREG function, remains unknown and was the focus of these investigations. Exercise can communicate with TREG cells by their expression of β2-adrenergic receptors (β2-AR). Activation of these receptors results in an increase in intracellular levels of cyclic-AMP, potentially creating a potent inhibitor of Th cell responses.

**Results:**

For the allergic asthma model, female wildtype BALB/c mice were challenged with OVA, and exercised (13.5 m/min for 45 min) 3×/week for 4 weeks. TREG cells were isolated from all mouse asthma/exercise groups, including β2-AR^−/−^ mice, to test suppressive function and intracellular cAMP levels. In these studies, cAMP levels were increased in TREG cells isolated from exercised mice. When β2-AR expression was absent on TREG cells, cAMP levels were significantly decreased. Correlatively, their suppressive function was compromised. Next, TREG cells from all mouse groups were tested for suppressive function after treatment with either a pharmaceutical β2-adrenergic agonist or an effector-specific cAMP analogue. These experiments showed TREG cell function was increased when treated with either a β2-adrenergic agonist or effector-specific cAMP analogue. Finally, female wildtype BALB/c mice were antibody-depleted of CD25^+^CD4^+^ TREG cells (anti-CD25). Twenty-four hours after TREG depletion, either β2-AR^−/−^ or wildtype TREG cells were adoptively transferred. Recipient mice underwent the asthma/exercise protocols. β2-AR^−/−^ TREG cells isolated from these mice showed no increase in TREG function in response to moderate aerobic exercise.

**Conclusion:**

These studies offer a novel role for β2-AR in regulating cAMP intracellular levels that can modify suppressive function in TREG cells.

## Background

T regulatory (TREG; Foxp3^+^CD4^+^CD25^+^) cells play a critical role in maintaining homeostasis of the cellular immune responses so as to prevent chronic inflammatory and autoimmune diseases [1]. Specifically, it has been shown that the balance between T helper effector (Th; CD4^+^CD25^−^) and TREG cells is essential for the proper control of adaptive immune responses. Therefore, understanding the mechanisms that determine this balance at both a cellular and molecular level holds promise for establishing novel immune intervention therapies in patients with chronic inflammatory and autoimmune diseases.

The suppressive/regulatory effects of TREG cells on Th cells can be mediated by contact-independent as well as contact-dependent mechanisms. Contact-independent mechanisms rely on the release of soluble factors, such as inhibitory cytokines (IL-10, TGF-β) and cytolytic molecules (granzymes), while cell contact-dependent mechanisms rely on cellular interactions that decrease antigen presentation between Th cells and antigen presenting cells (CTLA-4, LAG-3) or metabolic disruption of Th cell function (IL-2, cAMP) [1, 2]. Specific to the role of cAMP in TREG suppressive mechanisms, there are two sources of cAMP that contribute to Th cell suppression: extracellular and intracellular cAMP. Studies show TREG cells support the increased production of extracellular cAMP (CD39 and CD73) that can bind to receptors on Th cells and decrease activation [3, 4]. However, emerging evidence now suggests TREG cells can generate significant increases in intracellular cAMP that can be delivered to Th cells via gap junctions (GJIC) and will also result in decreased Th cell activation [5].

Our previous studies revealed an increase in contact-dependent TREG suppressive function in Th:TREG cell co-cultures in which TREG cells were isolated from aerobically exercised mice [6]. Schwiebert et al. showed that the exercise-induced increase in TREG function was sufficient to alleviate the characteristic symptoms of asthma pathogenesis: chronic airway inflammation and decreased lung function using an ovalbumin-driven murine asthma model [6, 7]. Because these experiments showed TREG cells isolated from exercised mice demonstrate an increase in TREG suppressive function during a Th:TREG cell in vitro co-culture that was contact-dependent and cytokine-independent, we focused these investigations on the role of intracellular cAMP as a TREG regulatory mechanism.

During physical activity, epinephrine is secreted into the bloodstream by activating the sympathetic nervous system (SNS; fight or flight response). Epinephrine changes target cell function by binding to adrenergic receptors expressed on the cell surface. Exercise communicates with TREG cells, directly, by their expression of β2-adrenergic receptors (β2-AR) [8]. It is well established that in the presence of activated β2-ARs there is an increase in intracellular cAMP levels.

Intracellular cAMP-mediated signaling pathways regulate a multitude of important biological processes. The effects of cAMP are mediated by two ubiquitously expressed intracellular cAMP effector molecules: protein kinase A (PKA) and, the more recently acknowledged, exchange protein directly activated by cAMP/cAMP-regulated guanine nucleotide exchange factors (EPAC/cAMP-GEF). Significant research has shown high intracellular levels of cAMP in Th cells results in a potent inhibition of Th effector cell activation by both PKA-activated and EPAC-activated signaling pathways [5, 9–11]. However, less is known about intracellular cAMP signaling pathways in TREG cells. Studies by T. Bopp, J. Bodor and others suggest a novel role for cAMP in the quantification of TREG suppressive function [12]. They propose TREG intracellular cAMP levels are directly proportional to TREG suppressive capacity. Further, they suggest a role for PKA in the mediation of TREG function [13].

Because cAMP can regulate a multitude of processes, intracellular cAMP levels are tightly regulated by a system of adenylate cyclases (AC) that form cAMP and phosphodiesterases (PDE) that breakdown cAMP. Studies by Umansky et al. and others show Th and TREG cells have distinct AC/PDE expression profiles. Notably, the differential expression of ACs and PDEs in TREG and Th cells explain the unique capacity to generate high intracellular cAMP signals in TREG cells while Th cells maintain low cAMP levels until after Th cell activation [14, 15].

The ability of TREG cells to accumulate and maintain high levels of intracellular cyclic-AMP allows for transfer of cAMP through membrane gap junctions (GJIC) to target cells such as Th effectors with low levels of cAMP [5, 11, 12]. GJIC channels are formed by a group of proteins called connexins. Connexin 43 (Cx43) is a fundamental component of the immune synapse between TREG and Th effector cells. In TREG cells, Cx43 has been shown to support FoxP3^+^ expression and to maintain a suppressive TREG phenotype [16]. Further, cAMP-activated PKA pathways have been shown to enhance GJIC assembly by increasing organized Cx43 interactions with the cytoskeleton and other signaling molecules [17]. Additionally, TREG cells isolated from FoxP3^−/−^ mice indicated significant loss of suppressive function with correlative decreases in intracellular cAMP levels [18]. Taken together, these studies further support Bodor’s proposed role for cAMP in the quantification of TREG suppressive function and our hypothesis in these investigations. Specifically, can exercise increase β2-adrenergic receptor activation to increase intracellular cAMP levels that correlates to the increased TREG suppressive function. Ultimately, these experiments sought to understand why moderate aerobic exercise can increase TREG suppressive function in a contact-dependent manner as previously described by Lowder et al. [6, 7].

In these presented studies, we show that exercise does increase intracellular cAMP levels that correlatively increase TREG suppressive function. Further, we show TREG cells lacking β2-adrenergic receptor expression indicate decreased cAMP levels that correlatively decrease suppressive function. Additionally, TREG culture with a pharmaceutical β2-adrenergic agonist is sufficient to gain suppressive function in TREG cells of all mouse treatment groups, exercised or sedentary, OVA-sensitized or non-sensitized. For the first time, we show that cAMP analogues that activate EPAC1 in TREG cells shows a greater increase in modifying suppressive function than cAMP analogues specific to PKA activated pathways. Finally, mice that contain β2-AR^−/−^ TREG cells lose responsiveness to moderate aerobic exercise showing no gain in TREG suppressive function in vivo. These results suggest a novel role for β2-ARs in maintaining TREG suppressive capabilities.

## Methods

### Mice

Six to eight week-old female BALB/c (Taconic) were used for all recipient and wild type experiments. Age-matched β2-AR^−/−^ BALB/c (from Dr. Virginia Sanders, Ohio State University managed Taconic colony, with permission from Dr. Kobilka, ( [19, 20])) were used for in vitro and adoptive transfer (ADTX) experiments as indicated. All mice were maintained in autoclaved microisolator cages (Lab Products) and provided with food (Teklad) and water, as needed. All mice were housed in the University of South Alabama, College of Medicine, Animal Care Facility. At the end of each protocol, mice were terminated using 1) overdose of chemical anesthetics (ketamine 80 mg/kg:Xylazine 10 mg/kg-- 2-3 times the anesthetic dose) to loss of toe pinch and corneal reflex and, either 2) exsanguination by intracardiac puncture or 3) vital tissue/organ collection (removal of lungs) as per IACUC approval (*USA - IACUC 346362–4*).

### OVA-sensitization and challenge

**Fig. 1:**
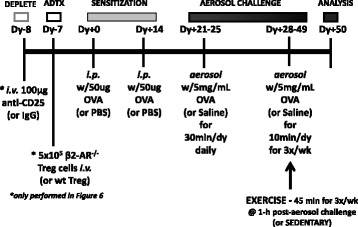
TREG depletion/OVA – sensitization and challenge/exercise training timeline. BALB/c mouse experimental groups (Figs. 2-5): (a) Sedentary non-sensitized (S); (b) Exercised non-sensitized (E); (EO); (c) Sedentary OVA-sensitized (SO); (d) Exercised OVA-sensitized. Briefly, OVA-sensitized (SO and EO) mice were intraperitoneally (i.p.) injected with 50 μg of alum-precipitated, chicken egg OVA (or PBS for non-sensitized - S and E) on Days + 0 and + 14 [**antigen SENSITIZATION**]. On Days + 21–25 [**AEROSOL CHALLENGE**], OVA-sensitized mice (SO and EO) were challenged with OVA at 5 mg/mL (or saline for non-sensitized – S and E) via 30 min daily aerosolization for 5 consecutive days. On Days + 28–49 [**AEROSOL CHALLENGE**], OVA-sensitized mice (SO and EO) were further challenged with OVA at 5 mg/mL (or saline for non-sensitized – S and E) for 10 min at 3 doses/week. Additionally, on Days + 28–49 [**EXERCISE**], exercised mouse groups (E and EO) were placed on a treadmill for a moderate aerobic exercise (45 min at 13.5 m/min) one hour after the aerosol challenge. (*) In experiments that required **TREG depletion** (Fig. 6), on Day − 8, mice were initially intravenously (i.v.) injected with 100 μg anti-CD25 mAb (PC61.5) or control IgG mAb. On Day − 7 [**ADTX**], 24 h depleted mice received either 5 × 10^5^ wildtype BALB/c or β2-adrenergic^−/−^ BALB/c CD4^+^CD25^+^ TREG adoptively transferred cells by i.v. Additional mouse experimental groups: (a) Sedentary OVA-sensitized – IgG control (SO-IgG); (b) Exercised OVA-sensitized – IgG control (EO-IgG); (c) Sedentary OVA-sensitized – WT ADTX (SO-wt) (d) Exercised OVA-sensitized – WT ADTX (EO-wt); (e) Sedentary OVA-sensitized – β2-AR^−/−^ ADTX (SO-ko) (d) Exercised OVA-sensitized – β2-AR^−/−^ ADTX (EO-ko)

Mice were randomly assigned to four experimental groups: (a) Sedentary non-sensitized (S); (b) Exercised non-sensitized (E); (c) Sedentary OVA-sensitized (SO); (d) Exercised OVA-sensitized (EO). OVA-sensitized mice (SO and EO) were challenged with ovalbumin (OVA) as previously described [7]. Briefly, OVA-sensitized (SO, EO) mouse groups were injected intra-peritoneal (i.p.) with 200 μL of 0.25 μg/μL(50 μg total) alum-precipitated, chicken egg ovalbumin (Imject Alum; Grade VII OVA ≥98% pure, Sigma Chemical) while non-sensitized controls (S and E) received an i.p. injection of PBS on Days + 0 and + 14. Beginning on Day + 21, OVA-sensitized mice (SO and EO) were exposed to aerosolized OVA at a concentration of 5 mg/mL in 0.9% w v^− 1^ NaCl solution (saline) while non-sensitized mouse groups (S and E) received saline alone for 30 min/day for five consecutive days (Days + 21–25). Aerosolized OVA [or saline] at the same concentration was administered for 10 min/day at 3 days/week for the remainder of the study (Days + 28–49) (see Fig. 1). This method of OVA-sensitization has been demonstrated previously to induce an allergic response as indicated by increases in serum OVA-specific IgE levels [7].

### Exercise training

Exercise training was performed 3 days/week for a total time period of 4 weeks for all exercise mouse groups (E and EO) (see Fig. 1). The first week of the exercise regimen consisted of a training and acclimation period whereby mice were subject to running beginning at 10.0 m/min for 30 min and gradually increased to 13.5 m/min for 45 min. In the remainder of the protocol, mice exercised (E and EO) for 45 min at 13.5 m/min (0% grade). All exercise sessions included brief warm-up and cool-down periods so that the total treadmill time was approximately 60 min. To avoid novel effects, the control sedentary mice (S and SO) were placed on the treadmill for 10 min without exercise. No stimulus (i.e. electric shock or other mechanical stimulus) was used in this study. Sedentary mice were housed in the same room and were subjected to all the same conditions, without the exercise-training component. All exercise sessions commenced one hour post-OVA aerosolization challenge on Days + 28–49 at 3 sessions/week frequency, as described above in the OVA sensitization protocol [7]. Exercise training during those times was executed 3 days/week for 10 sessions (see Fig. 1). Mice were sacrificed for analysis at 18 h after the final exercise session via ketamine:xylazine (ketamine 160 mg/kg:xylazine 20 mg/kg) overdose and subsequent exsanguination.

### TREG and T helper cell isolation

To isolate CD4^+^ CD25^+^ TREG cells, the spleens and lymph nodes of wild type or β2-AR^−/−^ mice were removed and a “single-cell suspension” was prepared. Red blood cells were depleted/lysed using a lysis solution, ACK buffer (Fisher Scientific). CD4^+^ T cells were isolated from the single-cell suspension using a magnetic bead “negative selection” separation kit (Miltenyi Biotec, Auburn, CA, USA), according to the manufacturer’s protocol. Briefly, CD8^+^ CD220^+^, CD11b^+^, DX5^+^, and Ter-119^+^ cells were depleted from the harvested cell population using biotin-labeled specific mAbs. Anti-biotin magnetic beads and an LD magnetic bead column were used to separate the biotin-tagged antibody-labeled cells from the harvested cell population. The “negatively isolated” CD4^+^ T cells were then “positively selected” for CD25 by incubating with Phycoerythrin (PE)-labeled anti-CD25 antibodies. Anti-PE magnetic beads and the MS column were used to isolate CD4^+^ CD25^+^ TREG cells. CD4^+^CD25^−^ T helper cells were collected from the run off. The purity of the sorted cells was > 90%.

### TREG cell suppression assay

**Fig. 2:**
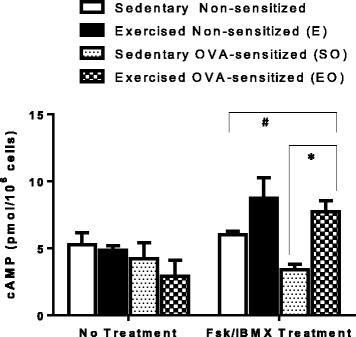
Moderate aerobic exercise increases TREG intracellular cAMP levels when treated with Fsk/IBMX. TREG cells were isolated from all experimental mouse groups (S, E, SO, WO). Cyclic-AMP levels were analyzed by RIA in lysed TREG cells either with or without forskolin and IBMX treatment. (*n* = 5 in triplicate) Data are shown as mean +/− SEM of triplicates for three independent experiments. *P < 0.05 between groups where indicated. # Two-way ANOVA: OVA treatment - n.s., Exercise treatment - *p* = 0.0071, Interaction - n.s

Wild type or β2-AR^−/−^ CD4^+^CD25^+^ TREG cells and wildtype CD4^+^CD25^−^ effector Th cells were isolated as previously described. In order to compare suppression function levels between treatment groups, CD4^+^CD25^−^ effector Th cells for all suppression assays were derived from OVA-treated, sedentary (SO) wildtype mice. CD4^+^CD25^+^ TREG cells (at increasing titers, Figs. 2 or 1:1 for all other figures) were co-cultured in complete RPMI 1640 (supplemented with glutamine/10% FBS/1% Pen-Strep/1% Pyruvate/50 μM 2-Mercaptoethanol) containing 5 μg/mL anti-CD3 (eBioscience) and 2 μg/mL anti-CD28 (eBioscience) with CD4^+^CD25^−^ effector Th cells. The Th cell population remained constant at 50,000 cells per well. Cells were cultured for 72 h (37 °C, 5% CO2). Tritiated-thymidine (3H, 0.5 μCi/well; Amersham) was added to all co-cultures in the last 18 h. Cultures were analyzed for effector Th cell proliferation by 3H incorporation via scintillation counting (PHD Harvester, BRANDEL).

### Determination of cyclic-AMP TREG cell lysates

Whole cell cAMP concentrations were determined using standard radioimmunoassay following the manufacturer’s instructions (Biomedical Technologies, Stoughton, MA). cAMP levels were normalized to protein content using the Micro Lowry with Peterson’s modification method (Sigma, St. Louis, MO). Prior to TREG cell cAMP analysis, positive controls were treated with forskolin (Fsk, 100 μM) and 3-isobutyl-1-methylxanthine (IBMX, 100 μM), a phosphodiesterase inhibitor. Cells were incubated for 30 min prior to cell lysis and cAMP analysis.

### In vivo CD4^+^CD25^+^ TREG cell depletion

To deplete CD4^+^CD25^+^ TREG cells in vivo, BALB/c mice were intravenously injected with 100 μg of anti-CD25 IgG mAb (PC61.5) or isotype control mouse IgG mAb (BD PharMingen, San Diego, CA). TREG (CD4 + CD25+) depletion was confirmed by flow cytometry in peripheral blood using anti-CD4 and CD25 mAb (7D4). Depleted TREG levels were comparable to other studies [21]. Twenty-four hours after TREG (CD4 + CD25+) depletion, mice received an adoptive transfer of 5 × 10^5^ CD4^+^CD25^+^ TREG cells collected from either wild type or β2-AR^−/−^ BALB/c mice, as described below (Fig. 1*).

### Adoptive transfer

BALB/c (Taconic) or β2-AR^−/−^ BALB/c (Taconic) mice were sacrificed to harvest spleen and lymph nodes for CD4^+^CD25^+^ TREG cells. Organs were homogenized and red blood cells lysed to yield a single cell suspension. The concentrations of cells were adjusted for 5 × 10^5^CD4^+^CD25^+^ TREG cells in 300 μL of no serum Dulbecco’s Modified Eagle’s Medium (DMEM). Each wildtype BALB/c recipient mouse received cells via intravenous (i.v.) injection in the tail vein while in a mouse restrainer on Day − 1 of the OVA-sensitization and challenge protocol (see Fig. 1*) [21].

### Bronchio-alveolar lavages

Mice were prepared for bronchio-alveolar lavage (BAL) as previously described [6]. Briefly, a catheter was inserted in the trachea of a terminally anesthetized mouse and 3 × 1 mL of buffered salt solution was gently inserted into and retrieved from the bronchioles.

### Analysis of cytokine levels

BAL fluid samples were analyzed for concentrations of mouse IL-4, IL-5, and IL-13 using commercial ELISA kits according to the manufacturer’s instructions (R&D Systems).

### Data analysis and statistics

Statistical significance between sedentary/exercised treatment and/or wildtype/β2-AR^−/−^ phenotypes during TREG suppression assay and cAMP level analysis was calculated using student’s T test, repeated measures ANOVA and/or two-way ANOVA. The effect of exercise on cAMP levels and suppressive function of TREG cells was analyzed comparing; exercised non-sensitized and sedentary non-sensitized or exercised-OVA sensitized and sedentary-OVA sensitized TREG populations. The effect of β2-AR presence on cAMP levels and suppressive function of TREG cells was analyzed comparing; wildtype and β2-AR^−/−^ TREG populations. The effect of pharmaceutical β2-AR activation on cAMP levels and suppressive function of TREG cells was analyzed comparing; wildtype untreated and wildtype β2-AR agonist treated TREG populations. The effect of cAMP analogue PKA and/or EPAC activation on suppressive function of TREG cells was analyzed comparing; exercised non-sensitized and sedentary non-sensitized with/without PKA activation; exercised non-sensitized and sedentary non-sensitized with/without EPAC activation; exercised-OVA sensitized and sedentary-OVA sensitized with/without PKA activation; or exercised-OVA sensitized and sedentary-OVA sensitized with/without EPAC activation of TREG populations. Each of these pair analyses used a paired student’s *t*-test for all single variable comparisons. In order to distinguish significant differences between cAMP and/or TREG function of the four experimental groups: (a) Sedentary non-sensitized (S); (b) Exercised non-sensitized (E); (c) Sedentary OVA-sensitized (SO); (d) Exercised OVA-sensitized (EO); data was analyzed using two-way ANOVA analysis between the two variables of exercise treatment and OVA treatment as indicated (GraphPad Prism 5). In order to distinguish significant differences between cAMP and/or TREG function of the experimental groups: [1] exercised OVA-sensitized wildtype ADTX (EO-wt); [2] sedentary OVA-sensitized wildtype ADTX (SO-wt); [3] exercised OVA-sensitized β2-AR^−/−^ADTX (EO-ko); and [4] sedentary OVA-sensitized β2-AR^−/−^ ADTX (SO-ko); data was analyzed using two-way ANOVA analysis between the two variables of exercise treatment and β2-AR presence as indicated (GraphPad Prism 5). Results were then reported as group means ±standard error of the mean (SEM) with significance set at a level of *p* ≤ 0.05 and interaction of variables.

## Results

### TREGs isolated from exercised mice show increased intracellular cAMP levels

In our previously published experiments, we show that moderate aerobic exercise (45 min at 13.5 m/min (0% grade) at 3×/week for 4 weeks) increased TREG suppression of artificially activated Th effector cells in vitro*.* Th effectors were isolated from mice undergoing an OVA-driven allergic asthma challenge protocol (see Fig. 1) [22]. In those studies, the exercise-induced increase in TREG suppression was cell contact dependent as indicated by experiments that showed no observable increase in TREG suppression of cells isolated from exercised mice when TREGs were co-cultured with Th cells using a transwell membrane cell culture system. Further, we concluded that the exercise-induced increase in TREG suppression was independent of cytokine production as indicated by experiments that continued to show an increase in suppressive function when TREGs isolated from exercised mice were co-cultured with Th cells in the presence of anti-IL-10 and/or anti-TGF-β. For these reasons, we investigated the contact-dependent TREG regulatory mechanism, intracellular cAMP, in exercised mice. Mice underwent exercise and OVA-sensitization protocols as indicated in Fig. 1. At the end of the protocol, TREG cells were magnetically isolated from all mouse groups (S, E, SO and EO) and assessed for intracellular cAMP levels by radioimmunoassay (RIA). No significant change in absolute cAMP levels were detected between mouse treatment groups of TREG cells (Fig. 2). However, because dynamic cAMP intracellular levels are tightly regulated by a series of adenylate cyclases and phosphodiesterase isoforms, we analyzed cAMP levels from TREG cells of all mouse treatment groups after exposure with forskolin (an activator of adenylate cyclases) and 3-isobutyl-1-methyl xanthine (IBMX, an inhibitor of phosphodiesterases). These experiments showed a notable increase in all exercised groups (E and EO) as compared to sedentary controls (S and SO) (Fig. 2). These findings show exercise can amplify cAMP signals in TREG cells. In order to exclude the role of OVA treatment in the observed intracellular cAMP increase, we performed a two-way ANOVA analysis. These statistical analyses indicated that exercise was the significant contributor for the differences observed in TREG cells isolated from either exercised or sedentary mice (OVA treatment - n.s., Exercise treatment - *p* = 0.0071, Interaction - n.s., *n* = 5–7 in triplicate).

### TREG cells lacking β2-adrenergic receptor expression show decreased cyclic-AMP levels that correlate with decreased suppressive function

**Fig. 3:**
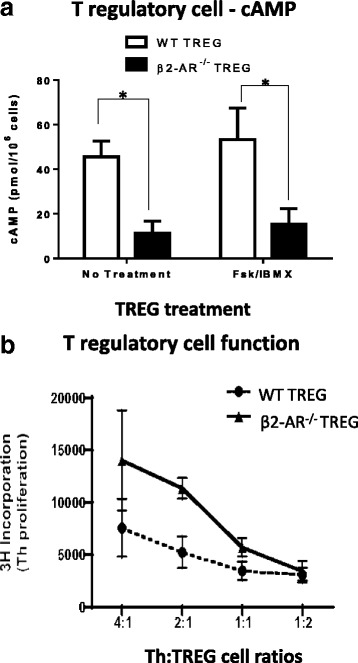
TREG cells lacking β2-AR expression exhibit decreased intracellular cAMP levels and TREG cell suppressive function. TREG cells (CD4^+^CD25^+^) with (WT TREG) or without (β2-AR^−/−^ TREG) β2-adrenergic receptors were negatively isolated and; (**a**) assessed for cyclic-AMP levels after lysing using RIA. (*n* = 5 in triplicate) *P ≤ 0.05 between groups where indicated. **b** co-cultured with wildtype CD4^+^CD25^−^ naive Th cells using artificial Th cell activation (anti-CD3 and anti-CD28) for 72 h. Th cell proliferation was assessed using 3H–thymidine incorporation. *P < 0.01 for repeated measures ANOVA analysis. All data are shown as mean +/− SEM of triplicates for three independent experiments

Exercise can communicate with TREG cells directly via β2-adrenergic receptor expression [8]. Because β2-adrenergic receptors are adenylate cyclase linked G-protein coupled receptors that produce cAMP upon stimulation, we investigated the role of β2-adrenergic receptors in maintaining intracellular cAMP levels within TREG cells. TREG cells were magnetically isolated from β2-AR^−/−^ mice and assessed for cAMP. Additionally, duplicate TREG cells (wildtype and β2-AR^−/−^) were treated with forskolin and IBMX. In both sets of experiments, TREG cells that lacked β2-adrenergic receptor expression showed significantly reduced cAMP levels when compared to wildtype TREG populations (Fig. 3a; *t*-test WT compared to β2-AR^−/−^, no treatment, *p* = 0.0081, fsk/IBMX, *p* = 0.05, *n* = 5–7 in triplicate). In order to determine whether the decrease in cAMP levels translated to decreased TREG suppressive function, β2-AR^−/−^ TREGs were co-cultured with naïve wildtype Th cells at ratios indicated on Fig. 3b. Th cells were artificially activated with anti-CD3 and anti-CD28 and assessed for Th cell proliferation. Notably, β2-AR^−/−^ TREG cells were unable to effectively suppress Th cell proliferation when compared to wildtype TREG cells (Fig. 3b; Repeated measures ANOVA – *p* < 0.01, *n* = 5–7 in triplicate). These findings show β2-adrenergic receptor expression on TREG cells contribute to intracellular cAMP levels. Further, these data indicate β2-adrenergic receptor expression is required for adequate TREG suppressive function.

### Pharmaceutical stimulation of β2-adrenergic receptors on TREG cells increases suppressive function

**Fig. 4:**
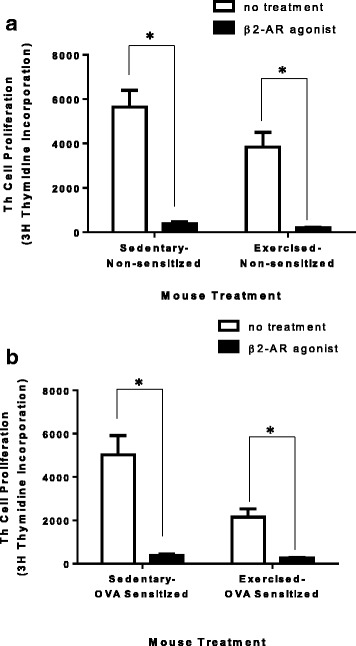
Treatment with formoterol is sufficient to increase TREG suppressive function. TREG cells isolated from all experimental mouse groups (S, E, SO, EO) were treated with a β2-adrenergic receptor agonist (formoterol) for 30 min. Formoterol-treated TREG cells were co-cultured with untreated non-sensitized naive Th cells using artificial Th cell activation (anti-CD3 and anti-CD28) for 72 h. Th cell proliferation was assessed using 3H–thymidine incorporation. **a** non-sensitized TREG cells (S and E). (*n* = 7–10) *P < 0.0001 between groups where indicated. Two-way ANOVA: β2-agonist treatment - *p* < 0.0001, Exercise treatment - *p* = 0.05, Interaction - n.s. or (**b**) OVA-sensitized TREG cells (SO and EO). (*n* = 7–10) *P < 0.0002 between groups where indicated. Two-way ANOVA: β2-agonist treatment - *p* < 0.0001, Exercise treatment - *p* = 0.0031, Interaction – *p* = 0.005. All data are shown as mean +/− SEM of triplicates of seven independent experiments

To substantiate these findings, we investigated whether pharmaceutically activating β2-adrenergic receptors on TREG cells would be sufficient to increase suppressive function of TREG cells isolated from non-sensitized sedentary mice. Additionally, could activating β2-adrenergic receptors on TREG cells further increase suppressive function of TREG cells after mice have undergone our OVA-sensitized and exercise protocols as indicated in Fig. 1. At the end of the protocol, TREG cells were magnetically isolated and treated with formoterol (long-acting β2-adrenergic agonist) for 30 min prior to 3× PBS washing of TREG cells. After washing, TREG cells were co-cultured with untreated wildtype sedentary Th cells. When TREG cells isolated from non-sensitized mice were treated with a β2-agonist, TREGs showed a significant increase in suppression of Th cell proliferation independent of exercise treatment (Fig. 4a; all significant pairings T-test *p* ≤ 0.0001; two-way ANOVA exercise *p* = 0.05, β-agonist *p* < 0.0001, interaction n.s., *n* = 7–10). When TREGs were isolated from OVA-sensitized mice and β2-adrenergic receptors were pharmaceutically activated with a long-acting beta-2 adrenergic agonist, TREGs showed a significant increase in suppression of Th cell proliferation that was additive to exercise treatment (Fig. 4B; all significant pairings T-test *p* ≤ 0.0002; two-way ANOVA exercise *p* = 0.0031 β-agonist *p* < 0.0001, interaction *p* = 0.0055, *n* = 7–10).

### Addition of cAMP effector-specific membrane permeable cAMP analogues to TREG cells increases suppressive function, although the EPAC-specific analogue increases TREG function more significantly than a PKA-specific cAMP analogue

**Fig. 5:**
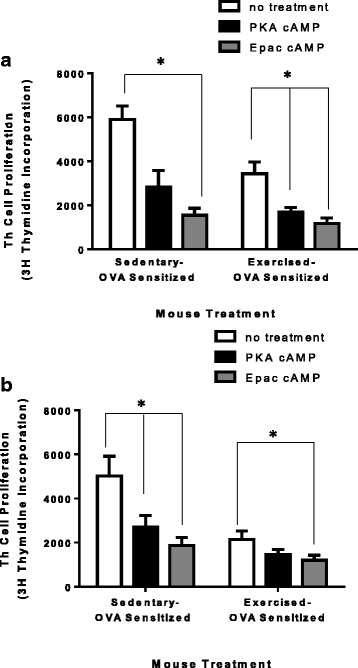
Treatment with effector specific membrane permeable cAMP analogues is sufficient to increase TREG suppressive function. TREG cells were treated with either PKA- or EPAC1- specific cyclic-AMP for 30 min. cAMP-treated TREG cells were co-cultured with untreated non-sensitized naive Th cells using artificial Th cell activation (anti-CD3 and anti-CD28) for 72 h. Th cell proliferation was assessed using 3H–thymidine incorporation. **a** non-sensitized TREG cells. (*n* = 7–10) *P ≤ 0.02 between groups where indicated. **b** OVA-sensitized TREG cells. (*n* = 7–10) *P ≤ 0.04 between groups where indicated. All data are shown as mean +/− SEM of triplicates of seven independent experiments

Because cAMP activates multiple pathways that control many cell functions, we investigated whether the activation of these cAMP pathways could increase TREG cell function. Specifically, cAMP activates signaling pathways primarily directed by either protein kinase A (PKA) or exchange protein directly activated by cAMP (EPAC). These experiments were aimed to determine whether one of these cAMP-activated effector molecules is more influential in increasing TREG suppressive function. Mice underwent the exercise and OVA-sensitization protocols (Fig. 1). At the end of this protocol, TREG cells were magnetically isolated from all mouse groups (S, E, SO, and EO) and treated for 30 min with either PKA-specific or EPAC-specific cell permeable effector-specific cAMP analogues. Treated TREG cells were washed 3× with PBS and co-cultured with untreated naïve wildtype Th cells. Th cells were artificially activated and measured for Th cell proliferation. In TREG cell populations isolated from non-sensitized mice, PKA-specific cAMP increased suppressive function significantly in the exercised cell populations when compared to no treatment but was unable to gain statistical significance in TREG populations from sedentary mice. Alternatively, EPAC-specific cAMP significantly increased TREG suppressive function in TREG populations isolated from either sedentary or exercised mice when compared with no treatment TREG groups (Fig. 5a; T-test, for all significant pairings *p* ≤ 0.02, *n* = 7–10). In the TREG populations isolated from OVA-sensitized mice, PKA-specific cAMP increased suppressive function significantly in the cell populations from sedentary mice when compared to TREG groups not treated with a cAMP analogue but did not gain statistical significance in the TREG cells isolated from exercised mice. In the OVA-sensitized TREG populations, EPAC-specific cAMP significantly increased TREG suppressive function in TREG populations from either sedentary or exercised mice when compared with TREG groups not treated with a cAMP analogue (Fig. 5b; T-test, for all significant pairings *p* ≤ 0.04, *n* = 7–10).

### TREG cells isolated from exercised mice do not show increased suppressive function when TREG cells do not express β2-adrenergic receptors

**Fig. 6:**
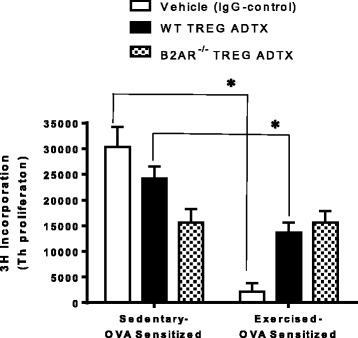
β2-AR^−/−^ TREG cells isolated from moderate aerobic exercised mice do not increase TREG suppressive function. Wildtype BALB/c mice were TREG cell depleted using anti-CD25 intravenous 24 h prior to receiving 5 × 10^5^ wildtype or β2-AR^−/−^ TREG cells. Mice underwent OVA-sensitization and exercise protocols (see Fig. 1). At the end of the protocol, TREG cells from each group were co-cultured with non-sensitized naive Th cells using artificial Th cell activation (anti-CD3 and anti-CD28) for 72 h. Th cell proliferation was assessed using 3H–thymidine incorporation. (*n* = 8–14) *P ≤ 0.005 between groups where indicated. Two-way ANOVA: exercise treatment – *p* = 0.04, β2-AR treatment – n.s., Interaction – *p* = 0.04. All data are shown as mean +/− SEM of triplicates of two independent experiments

Finally, we wanted to test whether β2-AR on TREGs played a significant role in how moderate aerobic exercise modified TREG function in vivo. Wildtype BALB/c mice were antibody depleted (anti-CD25) of TREG cells prior to receiving an adoptive transfer (ADTX) of either wildtype or β2-AR^−/−^ TREG cells as indicated in Fig. 1(*). Mice underwent OVA-sensitization and Exercise protocols. At the end of the protocol, TREG cells were isolated from all mouse groups (IgG control –EO and SO, WT ADTX – EO and SO, β2-AR^−/−^ ADTX – EO and SO) and co-cultured with naïve wildtype Th cells at a 1:1 ratio. In this experiment, we showed exercise is capable of increasing TREG suppressive capabilities in both IgG controls and the WT TREG ADTX cell groups when compared to their sedentary OVA-sensitized controls. Notably, mice that received β2-AR^−/−^ TREG cells and underwent exercise treatment were unable to modify TREG suppressive functions as compared to their sedentary control (Fig. 6; T-test, all significant pairings *p* ≤ 0.005; two-way ANOVA exercise *p* = 0.04, β2-AR n.s., interaction *p* = 0.04, *n* = 8–14).

### BAL fluid samples isolated from exercised mice do not show decreased Th2 cytokines as compared to sedentary controls when TREG cells do not express β2-adrenergic receptors

**Fig. 7:**
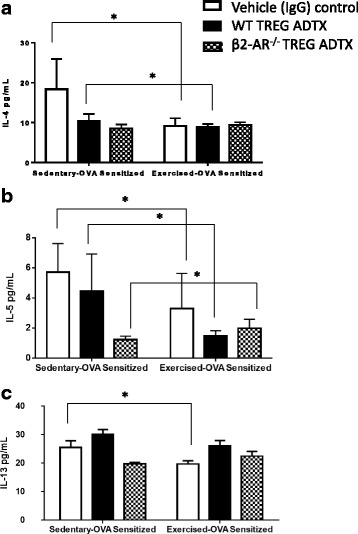
Th2 cytokines are not attenuated in moderately exercised mice after β2-AR^−/−^ TREG cell adoptive transfer. Wildtype BALB/c mice were TREG cell depleted using anti-CD25 intravenous 24 h prior to receiving 5 × 10^5 ^wildtype or β2-AR^−/−^ TREG cells. Mice underwent OVA-sensitization and exercise protocols (see Fig. 1). At the end of the protocol, bronchio-aveolar lavage fluid samples were collected from each mouse group and assessed for Th2 cytokine levels: (**a**) IL-4, Two-way ANOVA for IL-4: exercise treatment – *P* = 0.002, TREG treatment – *P* = 0.007., Interaction – *P* = 0.0008. **b** IL-5, Two-way ANOVA for IL-5: exercise treatment – *P* = 0.005, TREG treatment – *P* = 0.0004., Interaction – *P* = 0.016. **c** IL-13, Two-way ANOVA for IL-5: exercise treatment – n.s., TREG treatment – *P* = 0.0005., Interaction – *P* = 0.05. Th2 cytokine levels were assessed using a standard ELISA. (*n* = 6–8) All data are shown as mean +/− SEM of triplicates of two independent experiments. *P ≤ 0.05 between groups using a student *t*-test where indicated

Finally, we examined whether β2-AR on TREGs played a significant role in how moderate aerobic exercise decreased Th2 cytokine levels within the bronchioles as previously described [6]. Wildtype BALB/c mice were antibody depleted (anti-CD25) of TREG cells prior to receiving an adoptive transfer (ADTX) of either wildtype or β2-AR^−/−^ TREG cells as indicated in Fig. 1(*). Mice underwent OVA-sensitization and Exercise protocols. At the end of the protocol, bronchio-aveolar lavage fluid was collected from all mouse groups (IgG control –EO and SO, WT ADTX – EO and SO, β2-AR^−/− ^ADTX – EO and SO) and assessed for Th2 cytokines: IL-4, IL-5, IL-13. In this experiment, we showed exercise is capable of decreasing all Th2 cytokines (IL-4, IL-5, and IL-13) in IgG controls when exercise mice are compared to sedentary controls (Fig. 7a-c; T- test, all significant pairings *p* ≤ 0.05, *n* = 6–8). While assessing WT TREG ADTX mouse groups, exercise was able to significantly decrease IL-4 and IL-5 levels when compared to their sedentary controls (Fig. 7a-c; T-test, all significant pairings *p* ≤ 0.05, *n* = 6–8). However, there was no significant change in IL-13 levels of these WT TREG ADTX mouse groups. Next, mice that received β2-AR^−/−^ TREG cells and underwent exercise treatment showed no change in IL-4 and IL-13 cytokine levels as compared to their sedentary controls. Notably, when assessed for IL-5 levels, β2-AR^−/−^ TREG ADTX sedentary controls showed significantly increased IL-5 levels when compared to the exercised experimental β2-AR^−/−^ TREG ADTX mouse groups (Fig. 7b; T-test, IL-5 significant pairing *p* = 0.004, *n* = 6–8).

## Discussion

In our previous studies, we showed moderate-intensity aerobic exercise enhances/stabilizes TREG suppressive function within a chronic inflammatory microenvironment [6]. We observed that the enhanced TREG regulatory mechanism was contact-dependent in an in vitro co-culture of Th effector and TREG cells. In these earlier investigations, considerable effort was made to understand the mechanism to which TREG function was enhanced, but how exercise altered TREG function remained poorly understood. In this study, we demonstrate a role for β2-adrenergic receptor-activated elevations in cAMP as a key component in modulating the increase in suppressive function of TREG cells during moderate aerobic exercise.

A connection between cAMP levels and TREG cell function was first described by T. Bopp and colleagues showing high intracellular cAMP levels in murine TREGs are critically involved in contact-dependent suppression of Th effector cells [12, 23]. High intracellular levels of cAMP are transferred to Th effector cells via membrane gap junctions (GJICs) from TREG cells in order to suppress multiple Th cell activation and differentiation signaling pathways [5, 11, 12, 17]. To this end, cyclic-AMP production/breakdown is a highly dynamic process and tightly regulated by a variety of adenylate cyclases (ACs) and phosphodiesterases (PDEs) activity and/or expression within a cell. In this study, we show TREG cells isolated from exercised mice (E and EO) present with comparable levels of cAMP when compared to sedentary controls (S and SO). However, with TREG treatment with forskolin/IBMX, we show TREG cells isolated from exercised mice (E and EO) present significant increases in cAMP production capabilities when compared to sedentary controls (S and SO) (Fig. 2). This data is consistent with the notion that moderate aerobic exercise affects TREG signaling pathways that facilitate the increase in either activity or expression levels of adenylate cyclases and/or phosphodiesterases that regulate intracellular cAMP levels [24–27]. How AC/PDE activity and/or expression is altered in TREG cells is not well defined and will be the focus of further investigations.

Despite notable studies correlating cAMP to TREG suppressive potency, the specific sources of intracellular cAMP in TREG cells remains elusive. These experiments provide one mechanism in which TREG cAMP levels and subsequent cell function can be modulated by exercise and the activation of the sympathetic nervous system (SNS; fight or flight response). Exercise promotes the release of both glucocorticoids (GCs) and epinephrine in amounts proportional to intensity and duration. Specific to these studies, exercise increases systemic circulating epinephrine levels. Target cells expressing adrenergic receptors undergo cell function changes based on their receptor-signal molecule coupling. The β_2_-adrenergic receptor (β2-AR) has been shown to be the main adrenergic receptor expressed on immune cells including; dendritic cells, macrophages, CD4^+^ Th cells, CD8^+^ Tc cells, and B cells [28, 29]. β2-AR coupled adenylate cyclase activation generates a notable and transient cAMP intracellular signal. Recently, it was shown that CD4^+^CD25^+^ TREG cells also express β2-ARs [8].

The current studies suggest a role for β2-AR activation in the substantial accumulation of intracellular cAMP levels in TREG cells. TREG cells that do not express β2-AR (β2-AR^−/−^ TREGs) demonstrated a significant decrease in cAMP levels when compared to wildtype controls (WT TREGs) with and without Fsk/IBMX treatment (Fig. 3a). Further, these studies implicate β2-ARs in a maintenance role for sustaining basal intracellular cAMP levels of TREG cells.

Additionally, our data similarly demonstrate the quantitative connection between intracellular cAMP levels and TREG cell function as described by Bopp et al. [12, 23]. There was a correlative loss in TREG suppressive function in β2-AR^−/−^ TREGs as compared to wildtype TREG cells as indicated in the decreased cAMP and decreased function (Fig. 3b). However, it is worth noting that at an increased TREG to Th cell ratio (2:1), β2-AR^−/− ^TREGs were capable of preventing Th cell proliferation comparable to WT TREGs. These data suggest that other redundant TREG suppressive mechanisms are able to compensate for the loss of the observed β2-AR effect on TREG suppression. Notably, pharmaceutical activation of β2-ARs on TREG cells generate an increase in cAMP (data not shown) and a correlative increase in TREG suppressive function (Fig. 4a-b). The data presented in this manuscript indicating increased TREG function with treatment of a pharmaceutical β2-agonists further correlates with a study by Guereschi et al. using an alternate β2-agonist but yields the same results [8].

Notable to our studies, we show both sedentary and exercised TREG cells gain suppressive function with pharmaceutical activation of β2-ARs suggesting moderate aerobic exercise provides a modest stimulus, in both intensity and duration, for β2-ARs that can be further stimulated for additional gain of suppressive function (Fig. 4). Further, it may offer some understanding of why moderate exercise offers immune response benefits while exhaustive exercise can result in a compromised immune response [30–34].

Considerable progress has been made over the past decade elucidating how cyclic-AMP can inhibit T helper cell function after intercellular transfer of cAMP from TREG cells has occurred. Briefly, studies indicate a role for both PKA-dependent [35–38] and PKA-independent [39–43] signal transduction pathways that can participate in Th cell modifications. An increase in near-membrane pools of cAMP within Th cells activates PKA-driven kinases that result in T cell receptor (TCR) desensitization preventing further activation of Th cells [38, 44–50]. The cAMP-activated ICER transcription factor generates a prolonged deprivation of IL-2 gene transcription resulting in Th cell apoptosis [29, 51–53]. Despite tremendous research focused on cAMP inhibition of Th cell function; there is little knowledge on cAMP signaling targets and pathways within TREG cells, specifically. A 2013 publication suggested a PKA-dependent pathway within TREG cells [8]. However, our studies presented in this manuscript suggest an additional role for a PKA-independent pathway (EPAC1) as a modulator of suppressive function in TREG cells (Fig. 5a and b). The activation of the EPAC pathway showed a greater increase in TREG suppressive function than the activation of the PKA pathway. Therefore, both cAMP-activated pathways are implicated in facilitating the TREG regulatory mechanism indicated in a Th:TREG cell co-culture in vitro. Further, only the EPAC activated pathway significantly increased TREG suppression when isolated from exercised mice (E and EO) when compared to the PKA pathway activation in the same TREG cell populations. The challenge now will be to determine which of these actions of cAMP involve activation of the traditional protein kinase A (PKA)-regulated signal transduction pathway or the exchange protein directly activated by cAMP (EPAC)-regulated signal transduction pathway.

Discrete sub-cellular cAMP signals can activate specific PKA-dependent and/or PKA-independent intracellular signal cascades that modulate precise cell functions. As discussed earlier, significant research has identified these targets in Th effector cells [38, 44–51, 54–59], while in TREG cells these targets remain unclear. Further, these studies open up the possibility for novel investigations in EPAC-activation controlled cell functions of TREG cells.

Ultimately, we set out to define how moderate aerobic exercise increases TREG suppressive function subsequently decreasing Th2 cytokine expression and increased lung function, as we previously indicated in an ovalbumin-driven murine asthma model [6]. In Fig. 6, we demonstrated TREGs isolated from moderately exercised mice that expressed β2-AR (IgG control or WT TREG ADTX) were able to attenuate Th cell proliferation in an in vitro Th proliferation assay. Interestingly, TREGs isolated from β2-AR^−/− ^TREG ADTX exercised mice did not decrease Th cell proliferation as compared to the sedentary control. Thus, it is likely that moderate aerobic exercise increases TREG suppression function by the activation of β2-ARs on TREG that, in turn, generates high intracellular levels of cAMP to be transferred to Th effector cells in a contact- dependent manner to quantitatively increase TREG suppression. It is worth noting that in this experiment, TREGs isolated from β2-AR^−/−^ TREG ADTX mouse groups (exercised and sedentary) were able to adequately decrease Th proliferation to comparable levels. These data may suggest that in the absence of β2-AR, TREGs may rely on other redundant suppressive mechanisms that are unaffected by moderate aerobic exercise. However, these experiments do identify a novel role for β2-AR on TREG cells in altering suppressive function in response to a moderate aerobic exercise regimen that is capable of attenuating Th2 cytokines and, subsequently increasing lung function in a ovalbumin (OVA)-driven allergic asthma mouse model.

## Conclusions

In summary, we have demonstrated that moderate aerobic exercise alters the AC/PDE activity and/or expression levels to facilitate an increase in intracellular cAMP levels in TREG cells. Elevation of cAMP in TREG cells is quantitative and correlative to TREG suppressive function when measuring contact-dependent TREG regulatory mechanism of action. Increased cAMP by activation of β2-ARs is sufficient to increase TREG suppression. Correlatively, the absence of β2-ARs results in a significant decrease in cAMP and subsequent decrease in TREG suppressive function. Moderate aerobic exercise treatment requires β2-AR expression for accumulation of intracellular cAMP levels and the subsequent increase in TREG suppression.

These studies did not address downstream functions of these cAMP activated effector targets (PKA and EPAC) but did identify a novel role for β2-adrenergic receptor stimulated cAMP signals in maintaining TREG suppressive function. While significant progress has been made in understanding how cAMP suppresses Th function and enhances TREG function, the specific contribution by which β2-adrenergic receptor activation on TREG cells can modulate sub-cellular cAMP levels remains poorly understood.

## Abbreviations

AC: Adenylate cyclases
ADTX: Adoptive transfer
cAMP: Cyclic-AMP
Cx43: Connexin 43
E: Exercised non-sensitized
EO: Exercised OVA-sensitized
EO-ko: Exercised OVA-sensitized β2-AR^−/−^ ADTX
EO-wt: Exercised OVA-sensitized wildtype ADTX
EPAC/cAMP-GEF: Exchange protein directly activated by cAMP/cAMP-regulated guanine nucleotide exchange factors
Fsk: Forskolin
GJIC: Gap junction intercellular communication
IBMX: 3-isobutyl-1-methylxanthine
OVA: Ovalbumin
PDE: Phosphodiesterases
PKA: Protein kinase A
S: Sedentary non-sensitized
SNS: Sympathetic nervous system (fight or flight response)
SO: Sedentary OVA-sensitized
SO-ko: Sedentary OVA-sensitized β2-AR^−/−^ ADTX
SO-wt: Sedentary OVA-sensitized wildtype ADTX)
Th: T helper cells
TREG: T regulatory cells, CD4^+^CD25^+^
β2-AR: β2-adrenergic receptor
